# Is there a sodium effect in fibrillar amyloid-β oligomers?

**DOI:** 10.1186/1758-2946-6-S1-P56

**Published:** 2014-03-11

**Authors:** Anselm HC Horn, Danyil Huraskin, Heinrich Sticht

**Affiliations:** 1 Bioinformatik, Institut für Biochemie, Friedrich-Alexander-Universität Erlangen-Nürnberg, Fahrstr. 17, 91054 Erlangen, Germany

## 

Hall mark in Alzheimer’s Disease (AD) is the aggregation of amyloid-β (Aβ) peptide into oligomers and fibrils. Nowadays, the soluble oligomers are believed to be the most neurotoxic species, probably the causative agents in AD. Amyloid-beta fibrils on the other hand may serve as reservoirs for small toxic oligomers. While it is well known from experiment, that the aggregation process is modulated by salt concentration in solution, the molecular details of the underlying interactions are not.

Salts occur ubiquitously in physiological environments and are known to have profound effects on the solubility of proteins (Hofmeister series). Monovalent alkali metal ions exhibit a more subtle effect on Aβ aggregation in experiment than doubly charged species [1,2].

In this contribution we investigate the presence of the so-called ‘sodium-effect’ in fibrillar Aβ oligomers. This effect was originally described for dendrimer micelles formation modulating the self-organization of amphiphilic carboxylates: Na+ forms bridging complexes with carboxylate groups, in contrast to K+ [3].

Molecular dynamics simulations for a systematic series of single and double layer fibrillar Aβ oligomers in aqueous 150mM salt solution provide insights about the stabilizing interactions between the cations and charged Aβ key residues (e.g. Glu22). Comparison with a previous computational study at low ion concentration [4] show similarities and differences on a structural level. Interestingly, ions access the Aβ water channel region via entry paths suggested previously [4].
